# 791. ICU Provider Overestimation of Ventilator-Associated Pneumonia Diagnostic Probability: An Opportunity for Diagnostic Stewardship

**DOI:** 10.1093/ofid/ofac492.052

**Published:** 2022-12-15

**Authors:** Nathaniel Soper, Owen Albin

**Affiliations:** University of Michigan, Ann Arbor, MI; University of Michigan Medical School, Ann Arbor, Michigan

## Abstract

**Background:**

Overdiagnosis of ventilator-associated pneumonia (VAP) is common in intensive care units (ICUs). Because VAP is a clinical diagnosis without an easily accessible confirmatory diagnostic test, understanding how ICU physicians practice diagnostic reasoning in suspected VAP is vital to informing diagnostic stewardship efforts. We aimed to assess the accuracy of ICU practitioner estimates of VAP diagnostic probability.

**Methods:**

We conducted a survey of physicians at Michigan Medicine ICUs. Providers were presented with clinical vignettes (Figure 1) in which mechanically ventilated patients developed clinical signs suggestive of VAP and then had radiographic/microbiologic tests performed. Providers were asked to estimate the probability of VAP before and after each presented clinical sign or test result, which were then used to impute provider-estimated likelihood ratios (pLRs) for each clinical sign and diagnostic test. Provider-estimated probabilities and LRs were compared to evidence-based VAP probabilities and LRs (eLRs) derived from systematic reviews and meta-analyses.

Survey Case Example.

**Results:**

Of 102 survey respondents, 54% felt moderately to extremely confident in their ability to accurately diagnose VAP. Providers significantly overestimated the pre and post-test probability of VAP in all clinical scenarios (provider-estimated VAP diagnostic probabilities are visualized using density plots relative to evidence-based diagnostic probability in Figure 2). Median pLRs for nearly all VAP clinical signs and diagnostic tests were also significantly higher than eLRs (Figure 3), most notably for isolated purulent endotracheal secretions (pLR 1.54 vs eLR 0.76, p< 0.01), positive chest radiograph (pLR 6.0 vs eLR 3.55, p< 0.01), and positive bronchoalveolar lavage culture (pLR 5.7 vs eLR 1.37, p< 0.01).

Density Plots of Provider-Estimated vs Evidence-Based Diagnostic Probability of Ventilator-Associated Pneumonia.

Provider-Estimated vs Evidence-Based Diagnostic Likelihood Ratios for Clinical Signs and Test Results Related to Ventilator-Associated Pneumonia.

**Conclusion:**

Physicians routinely overestimated the diagnostic probability of VAP and the positive LRs of possible VAP clinical signs, as well as VAP diagnostic radiographic and microbiologic tests. Educational initiatives addressing and calibrating ICU providers’ perceptions of VAP diagnostic probability warrant investigation as an antimicrobial stewardship tool.

**Disclosures:**

**Owen Albin, MD**, Charles River Laboratory: Advisor/Consultant|Cipla Pharmaceuticals: Advisor/Consultant|Shionogi Inc: Advisor/Consultant.

**Figure 1.**
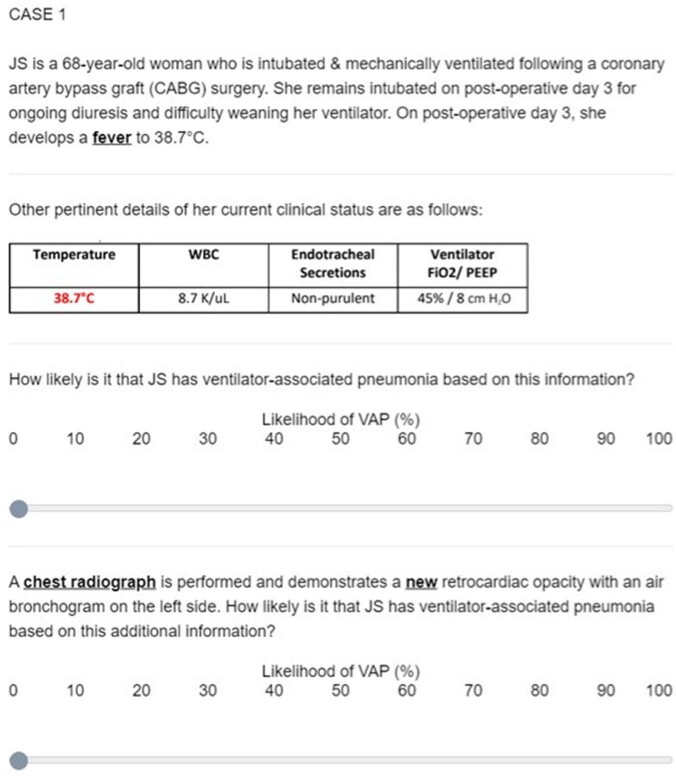
Excerpt from the digital survey showing an example of a clinical vignette. Respondents were presented with a clinical sign and a subsequent test result and were prompted to estimate the probability of VAP at each step.

**Figure 2.**
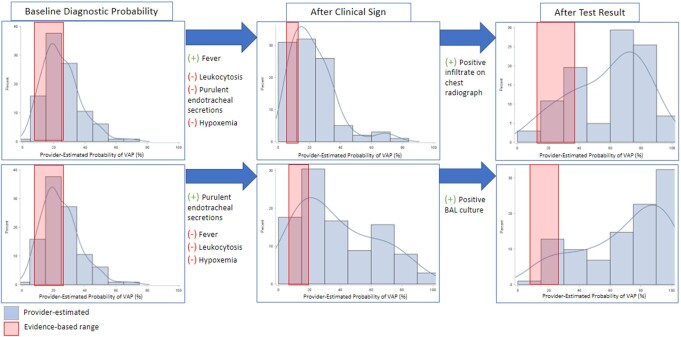
Density plots depicting provider-estimated diagnostic probability of VAP at baseline, after development of an isolated clinical sign, and following a subsequent test result. Provider-estimated probabilities are compared to evidence-based probabilities calculated using positive and negative likelihood ratios derived from VAP systematic reviews and meta-analyses. VAP = Ventilator-associated pneumonia. BAL = bronchoalveolar lavage.

**Figure 3.**
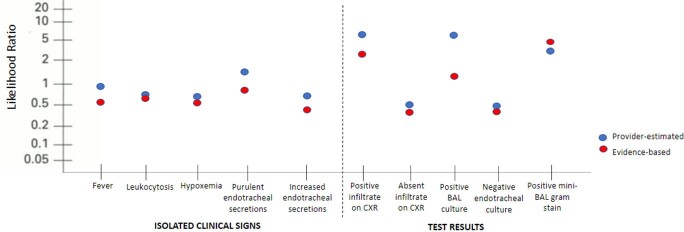
Scatterplot showing the differences in median provider-estimated diagnostic likelihood ratios relative to evidence-based likelihood ratios. Evidence-based likelihood ratios for an isolated clinical sign reflect the positive likelihood ratio of the stated clinical sign combined with the negative likelihood ratios of the remaining absent clinical signs. Provider-estimated likelihood ratios are calculated using survey responses for pre- and post-test probabilities. CXR = chest radiograph. BAL = bronchoalveolar lavage. "Positive infiltrate on CXR" refers to a chest radiograph with an opacity with an air bronchogram.

